# 1,3-Bis(4-*tert*-butyl­benz­yl)-4,5-dihydro­imidazolium chloride monohydrate

**DOI:** 10.1107/S1600536809000452

**Published:** 2009-01-14

**Authors:** Hakan Arslan, Don VanDerveer, Sedat Yaşar, İsmail Özdemir, Bekir Çetinkaya

**Affiliations:** a Department of Natural Sciences, Fayetteville State University, Fayetteville, NC 28301, USA; b Department of Chemistry, Faculty of Pharmacy, Mersin University, Mersin, TR 33169, Turkey; c Department of Chemistry, Clemson University, Clemson, SC 29634, USA; d Department of Chemistry, Faculty of Science and Arts, nönü University, Malatya, TR 44280, Turkey; e Department of Chemistry, Faculty of Science, Ege University, Bornova-zmir, TR 35100, Turkey

## Abstract

In the title compound, C_25_H_35_N_2_^+^·Cl^−^·H_2_O, the imidazolidine ring adopts a twisted conformation, with a pseudo-twofold axis passing through the N—C—N carbon and the opposite C—C bond. The N—C—N fragment of the imidazolidine ring shows some degree of both double- and single-bond character due to partial electron delocalization. One of the *tert*-butyl groups is disordered over two conformations with occupancies of 0.714 (8) and 0.286 (8). In the crystal, O—H⋯Cl and C—H⋯O hydrogen bonds help to establish the packing.

## Related literature

For synthesis, see: Yaşar *et al.* (2008 ▶); Özdemir *et al.* (2004*a* ▶, 2004*b* ▶). For general background, see: Herrmann *et al.* (1998 ▶); Glorius (2007 ▶); Nolan (2006 ▶). For related compounds, see: Arslan *et al.* (2009*a* ▶,*b* ▶) and references therein. For bond-length data, see: Allen *et al.* (1987 ▶). For puckering and asymmetry parameters, see: Cremer & Pople (1975 ▶); Nardelli (1983 ▶).
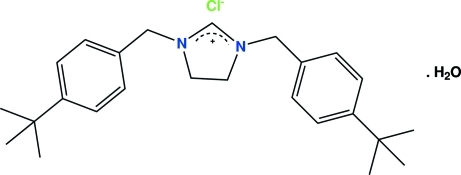


## Experimental

### 

#### Crystal data

C_25_H_35_N_2_^+^·Cl^−^·H_2_O
*M* *_r_* = 417.02
Monoclinic, 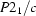

*a* = 18.205 (4) Å
*b* = 10.148 (2) Å
*c* = 13.452 (3) Å
β = 100.01 (3)°
*V* = 2447.3 (9) Å^3^
*Z* = 4
Mo *K*α radiation
μ = 0.17 mm^−1^
*T* = 153 (2) K
0.43 × 0.17 × 0.05 mm

#### Data collection

Rigaku AFC 8S Mercury CCD diffractometer
Absorption correction: multi-scan (*REQAB*; Jacobson, 1998 ▶) *T*_min_ = 0.929, *T*_max_ = 0.991
17800 measured reflections
4297 independent reflections
2886 reflections with *I* > 2σ(*I*)
*R*_int_ = 0.072

#### Refinement

*R*[*F* ^2^ > 2σ(*F* ^2^)] = 0.069
*wR*(*F* ^2^) = 0.207
*S* = 1.03
4297 reflections
299 parameters
50 restraints
H atoms treated by a mixture of independent and constrained refinement
Δρ_max_ = 0.28 e Å^−3^
Δρ_min_ = −0.26 e Å^−3^

### 

Data collection: *CrystalClear* (Rigaku/MSC, 2006 ▶); cell refinement: *CrystalClear*; data reduction: *CrystalClear*; program(s) used to solve structure: *SHELXTL* (Sheldrick, 2008 ▶); program(s) used to refine structure: *SHELXTL*; molecular graphics: *SHELXTL*; software used to prepare material for publication: *SHELXTL*.

## Supplementary Material

Crystal structure: contains datablocks global, I. DOI: 10.1107/S1600536809000452/hg2460sup1.cif

Structure factors: contains datablocks I. DOI: 10.1107/S1600536809000452/hg2460Isup2.hkl

Additional supplementary materials:  crystallographic information; 3D view; checkCIF report

## Figures and Tables

**Table 1:** Hydrogen-bond geometry (Å, °)

*D*—H⋯*A*	*D*—H	H⋯*A*	*D*⋯*A*	*D*—H⋯*A*
O1—H1*A*⋯Cl1^i^	0.86 (4)	2.36 (4)	3.206 (3)	174 (4)
C3—H3*B*⋯O1^ii^	0.96	2.53	3.304 (4)	138
C17—H17⋯O1^i^	0.96	2.47	3.423 (5)	175

Symmetry codes: (i) 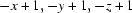; (ii) 
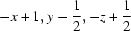
.

**Fig. 1.**
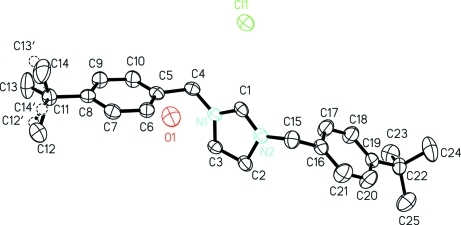
The molecular structure of the title compound, showing the atom-numbering scheme and displacement ellipsoids drawn at the 50% probability level.

**Fig. 2.**
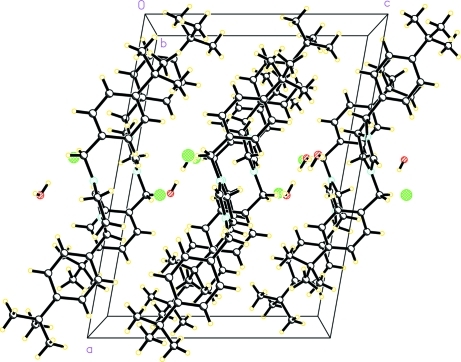
A packing diagram for (I).

**Fig. 3.**
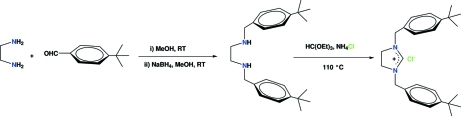
The formation of the title compound.

## supplementary crystallographic information

### Comment

The first report of the application of *N*-heterocyclic carbenes compounds to various reactions was in 1998 (Herrmann *et al.*, 1998). The metal complexes of *N*-heterocyclic carbene ligands have revealed excellent catalytic properties in a wide range of metal-catalyzed transformations such as Heck, Suzuki and Sonogashira couplings, Buchwald Hartwig amination, and olefin metathesis (Glorius, 2007; Nolan, 2006).

The use and characterization of an *in situ* formed imidazolidin-2-ylidene, tetrahydropyrimidin-2-ylidene, and the tetrahydrodiazepin-2-ylidene/palladium(II) system, which exhibits high activity in various coupling reactions of aryl bromides and aryl chlorides has been previously reported by our team (Yaşar *et al.*, 2008; Arslan *et al.*, 2009*a*, 2009*b*, and references therein). In order to obtain a more stable, efficient and active system, we have also investigated benzo-annelated derivatives (Özdemir *et al.*, 2004*a*, 2004*b*; Yaşar *et al.*, 2008). In the present work, we report the preparation and characterization of one of them, 1,3-*bis*(4-*tert*-butylbenzyl)-4,5-dihydroimidazolium chloride monohydrate, (I). The title compound was purified by re-crystallization from an ethanol:dichloromethane mixture (1:1) and characterized by elemental analysis, ^1^H and ^13^C-NMR and IR spectroscopy. The analytical and spectroscopic data are consistent with the proposed structure given in Scheme 1.

The molecular structure of the title compound is depicted in Fig. 1. The crystal structure is composed of a 1,3-*bis*(4-*tert*-butylbenzyl)-4,5-dihydroimidazolium cation, a Cl^-^ anion and a water molecule. The imidazolidine ring adopts a twisted conformation with a pseudo-twofold axis passing through C1 and the C2—C3 bond. The puckering parameters (Cremer & Pople, 1975) and the smallest displacement asymmetry parameter (Nardelli, 1983) for the imidazolidine ring are *q*_2_ = 0.073 (3) Å, *φ*_2 _= 306 (3)^o ^and Δ*C*_2_(C1) = 0.1 (3), Δ*C*_s_(C1) = 9.6 (3). The largest deviations from the mean plane of C1—N1—C3—C2—N2 ring are 0.044 (4) Å and 0.044 (3) Å for atom C2 and C3 atoms, respectively. One of the *t*-butyl groups is disordered over two conformations, with occupancies of 0.714 (8) and 0.286 (8).

The C—N bond lengths of N1—C1 and N2—C1 are shorter than the average single C—N bond length of 1.48 Å, being N1—C1 =1.319 (4) Å, N2—C1 = 1.312 (4) Å. The other C—N bond lengths (N1—C3 = 1.463 (4) Å, N2—C2 = 1.475 (4), N1—C4 = 1.458 (4) Å and N2—C15 = 1.449 (4) Å) are very close the average single C—N bond lengths. Therefore, we can easily say that the bonds in the N1—C1—N2 fragment of the imidazolidine ring are between double and single bond in character with partial electron delocalization. In addition, the sum of the bond angles around N1 (358.7°) and N2 (359.6°) indicate *sp*^2^ hybridization. The other bond lengths in (I) are in the normal ranges (Allen *et al.*, 1987).

The crystal packing is shown in Fig. 2. Although there are no intramolecular D—H···A contacts, intermolecular C—H···O and O—H···Cl hydrogen bonds link the molecules of (I) into one-dimensional chains extending along the [010] direction (Table 1).

### Experimental

The 4-*tert*-buthylbenzaldehyde (20 mmol) and the ethylenediamine (10 mmol) were stirred overnight in methanol. The diimine was collected as a white solid, filtrated and recrystallized in an alcohol/ether mixture. The diimine (10 mmol) was subsequently reduced by NaBH_4 _(30 mmol) in CH_3_OH (30 ml). The solution was then treated with 1 N HCl, and the organic phase was extracted with CH_2_Cl_2 _(3x30mL). After drying over MgSO_4_ and evaporation, the diamine was isolated as a solid. The diamine was then treated in a large excess of triethylorthoformate (50 ml) in the presence of 10 mmol of NH_4_Cl at 110 °C in a distillation apparatus until all of the ethanol was removed. Upon cooling to room temperature a colourless solid precipitated, which was collected by filtration and dried under vacuum. The crude product was recrystallized from absolute ethanol to give colourless needles and the solid was washed with diethyl ether (2x10 ml) and dried under vacuum. Yield: 3.53 g; 94%. *M*.p.: 294–295 °C; ν_(CN) _: 1669 cm^-1. 1^H NMR (δ, CDCl_3_): 1.24 (s, 18H, CH_2_C_6_H_4_(CC*H_3_*)_3_-4), 3.73 (s, 4H, NC*H*_2_CH_2_N), 4.80 (s, 4H, C*H*_2_C_6_H_4_(CCH*_3_*)_3_-4), 7.19–7.32 (m, 8H, CH_2_C_6_*H*_4_(CCH*_3_*)_3_-4), 10.20 (s, 1H, NC*H*N). ^13^C NMR (δ, CDCl_3_): 31.4 (CH_2_C_6_H_4_(C*C*H*_3_*)_3_-4), 34.9 (CH_2_C_6_H_4_(*C*CH*_3_*)_3_-4), 52.2 (*C*H_2_C_6_H_4_(CCH*_3_*)_3_-4), 126.3, 128.9, 129.8 and 152.3 (CH_2_*C*_6_H_4_(CCH*_3_*)_3_-4), 158.9 (N*C*HN). Anal. Calcd. for C_25_H_35_N_2_Cl: C,75.25; H, 8.84; N, 7.02%. Found: C, 75.20; H, 8.83; N, 7.0%.

### Refinement

All H atoms attached to carbons were geometrically fixed and allowed to ride on the corresponding non-H atom with C—H = 0.96 Å, and *U*_iso_(H) = 1.5*U*_eq_(C) of the attached C atom for methyl H atoms and 1.2*U*_eq_(C) for other H atoms. The water H atoms were located from a Fourier map and their distances were constrained to 0.83 Å and the *U*_iso_(H) = 1.5*U*_eq_(O). One of the *t*-butyl groups was disordered. Two components were refined with the major component having an occupancy of 0.714. Each component had constraints on the C11 - methyl distances (1.535 (50) Å) and the methyl-methyl distances (2.495 (50) Å). These values were obtained from the other *t*-butyl group in the structure.

### Crystal data

**Table:**

C_25_H_35_N_2_^+^·Cl^−^·H_2_O	*F*(000) = 904
*M**_r_* = 417.02	*D*_x_ = 1.132 Mg m^−^^3^
Monoclinic, *P*2_1_/*c*	Mo *K*α radiation, λ = 0.71073 Å
Hall symbol: -P 2ybc	Cell parameters from 5990 reflections
*a* = 18.205 (4) Å	θ = 2.3–26.7°
*b* = 10.148 (2) Å	µ = 0.17 mm^−^^1^
*c* = 13.452 (3) Å	*T* = 153 K
β = 100.01 (3)°	Plate, colorless
*V* = 2447.3 (9) Å^3^	0.43 × 0.17 × 0.05 mm
*Z* = 4	

### Data collection

**Table:**

Rigaku AFC 8S Mercury CCD diffractometer	4297 independent reflections
Radiation source: Sealed Tube	2886 reflections with *I* > 2σ(*I*)
Graphite Monochromator	*R*_int_ = 0.072
Detector resolution: 14.6306 pixels mm^-1^	θ_max_ = 25.0°, θ_min_ = 2.3°
ω scans	*h* = −15→21
Absorption correction: multi-scan (Jacobson, 1998)	*k* = −12→12
*T*_min_ = 0.929, *T*_max_ = 0.991	*l* = −16→16
17800 measured reflections	

### Refinement

**Table:**

Refinement on *F*^2^	Primary atom site location: structure-invariant direct methods
Least-squares matrix: full	Secondary atom site location: difference Fourier map
*R*[*F*^2^ > 2σ(*F*^2^)] = 0.069	Hydrogen site location: inferred from neighbouring sites
*wR*(*F*^2^) = 0.207	H atoms treated by a mixture of independent and constrained refinement
*S* = 1.03	*w* = 1/[σ^2^(*F*_o_^2^) + (0.0868*P*)^2^ + 3.6749*P*] where *P* = (*F*_o_^2^ + 2*F*_c_^2^)/3
4297 reflections	(Δ/σ)_max_ < 0.001
299 parameters	Δρ_max_ = 0.28 e Å^−^^3^
50 restraints	Δρ_min_ = −0.26 e Å^−^^3^

### Special details

**Table:**

Geometry. All e.s.d.'s (except the e.s.d. in the dihedral angle between two l.s. planes) are estimated using the full covariance matrix. The cell e.s.d.'s are taken into account individually in the estimation of e.s.d.'s in distances, angles and torsion angles; correlations between e.s.d.'s in cell parameters are only used when they are defined by crystal symmetry. An approximate (isotropic) treatment of cell e.s.d.'s is used for estimating e.s.d.'s involving l.s. planes.
Refinement. Refinement of *F*^2^ against ALL reflections. The weighted *R*-factor *wR* and goodness of fit *S* are based on *F*^2^, conventional *R*-factors *R* are based on *F*, with *F* set to zero for negative *F*^2^. The threshold expression of *F*^2^ > σ(*F*^2^) is used only for calculating *R*-factors(gt) *etc*. and is not relevant to the choice of reflections for refinement. *R*-factors based on *F*^2^ are statistically about twice as large as those based on *F*, and *R*- factors based on ALL data will be even larger.

### Fractional atomic coordinates and isotropic or equivalent isotropic displacement parameters (Å^2^)

**Table:**

	*x*	*y*	*z*	*U*_iso_*/*U*_eq_	Occ. (<1)
Cl1	0.55770 (5)	0.59372 (8)	0.69855 (6)	0.0407 (2)	
C1	0.44642 (19)	0.3585 (3)	0.5584 (2)	0.0329 (7)	
H1	0.4673	0.4424	0.5810	0.040*	
C2	0.3626 (2)	0.2034 (3)	0.4864 (3)	0.0408 (8)	
H2A	0.3513	0.1950	0.4143	0.049*	
H2B	0.3225	0.1667	0.5152	0.049*	
C3	0.43716 (19)	0.1369 (3)	0.5294 (2)	0.0348 (7)	
H3A	0.4319	0.0779	0.5835	0.042*	
H3B	0.4564	0.0890	0.4781	0.042*	
C4	0.56104 (19)	0.2349 (3)	0.6223 (2)	0.0358 (8)	
H4A	0.5607	0.1721	0.6756	0.043*	
H4B	0.5765	0.3181	0.6530	0.043*	
C5	0.61762 (19)	0.1916 (3)	0.5590 (2)	0.0328 (7)	
C6	0.61032 (19)	0.2201 (3)	0.4561 (2)	0.0362 (8)	
H6	0.5660	0.2627	0.4218	0.043*	
C7	0.6664 (2)	0.1874 (4)	0.4035 (3)	0.0402 (8)	
H7	0.6597	0.2071	0.3327	0.048*	
C8	0.73238 (19)	0.1271 (3)	0.4493 (3)	0.0360 (8)	
C9	0.7385 (2)	0.0989 (4)	0.5517 (3)	0.0422 (8)	
H9	0.7831	0.0576	0.5863	0.051*	
C10	0.6820 (2)	0.1288 (4)	0.6052 (3)	0.0433 (9)	
H10	0.6877	0.1055	0.6753	0.052*	
C11	0.7948 (2)	0.0960 (4)	0.3890 (3)	0.0421 (8)	
C12	0.7640 (4)	0.0329 (9)	0.2899 (5)	0.073 (2)	0.714 (8)
H12A	0.7393	−0.0478	0.3016	0.109*	0.714 (8)
H12B	0.8039	0.0146	0.2538	0.109*	0.714 (8)
H12C	0.7289	0.0916	0.2508	0.109*	0.714 (8)
C12'	0.7917 (13)	−0.0488 (16)	0.367 (2)	0.109 (9)	0.286 (8)
H12D	0.7449	−0.0699	0.3251	0.164*	0.286 (8)
H12E	0.7963	−0.0969	0.4293	0.164*	0.286 (8)
H12F	0.8319	−0.0724	0.3328	0.164*	0.286 (8)
C13	0.8549 (4)	0.0036 (8)	0.4466 (5)	0.072 (2)	0.714 (8)
H13A	0.8746	0.0411	0.5112	0.108*	0.714 (8)
H13B	0.8944	−0.0073	0.4084	0.108*	0.714 (8)
H13C	0.8331	−0.0807	0.4559	0.108*	0.714 (8)
C13'	0.8687 (10)	0.148 (3)	0.4447 (14)	0.109 (10)	0.286 (8)
H13D	0.8605	0.1968	0.5031	0.164*	0.286 (8)
H13E	0.8905	0.2053	0.4008	0.164*	0.286 (8)
H13F	0.9017	0.0759	0.4654	0.164*	0.286 (8)
C14	0.8337 (4)	0.2256 (7)	0.3712 (7)	0.072 (2)	0.714 (8)
H14A	0.8539	0.2650	0.4350	0.108*	0.714 (8)
H14B	0.7982	0.2847	0.3332	0.108*	0.714 (8)
H14C	0.8732	0.2084	0.3341	0.108*	0.714 (8)
C14'	0.7827 (11)	0.1702 (19)	0.2853 (13)	0.080 (6)	0.286 (8)
H14D	0.7322	0.1582	0.2516	0.121*	0.286 (8)
H14E	0.8162	0.1355	0.2440	0.121*	0.286 (8)
H14F	0.7923	0.2625	0.2967	0.121*	0.286 (8)
C15	0.3209 (2)	0.4448 (4)	0.4915 (3)	0.0431 (9)	
H15A	0.3039	0.4452	0.4198	0.052*	
H15B	0.3433	0.5288	0.5102	0.052*	
C16	0.2551 (2)	0.4253 (3)	0.5442 (3)	0.0377 (8)	
C17	0.2655 (2)	0.4019 (5)	0.6469 (3)	0.0528 (10)	
H17	0.3153	0.3980	0.6849	0.063*	
C18	0.2052 (2)	0.3841 (5)	0.6957 (3)	0.0532 (11)	
H18	0.2142	0.3690	0.7673	0.064*	
C19	0.1325 (2)	0.3875 (3)	0.6443 (3)	0.0382 (8)	
C20	0.1231 (2)	0.4119 (5)	0.5426 (3)	0.0550 (11)	
H20	0.0734	0.4161	0.5043	0.066*	
C21	0.1835 (2)	0.4307 (5)	0.4932 (3)	0.0549 (11)	
H21	0.1746	0.4478	0.4219	0.066*	
C22	0.0654 (2)	0.3722 (4)	0.6988 (3)	0.0459 (9)	
C23	0.0852 (3)	0.2921 (5)	0.7956 (3)	0.0615 (11)	
H23A	0.1047	0.2080	0.7805	0.092*	
H23B	0.0414	0.2794	0.8251	0.092*	
H23C	0.1221	0.3386	0.8424	0.092*	
C24	0.0407 (3)	0.5107 (5)	0.7246 (4)	0.0754 (15)	
H24A	−0.0034	0.5045	0.7543	0.113*	
H24B	0.0303	0.5626	0.6641	0.113*	
H24C	0.0798	0.5517	0.7714	0.113*	
C25	0.0003 (2)	0.3024 (5)	0.6312 (4)	0.0649 (12)	
H25A	0.0168	0.2187	0.6103	0.097*	
H25B	−0.0170	0.3557	0.5729	0.097*	
H25C	−0.0397	0.2892	0.6684	0.097*	
N1	0.48540 (15)	0.2483 (3)	0.5661 (2)	0.0333 (6)	
N2	0.37630 (16)	0.3421 (3)	0.5169 (2)	0.0353 (6)	
O1	0.55870 (15)	0.5901 (2)	0.2126 (2)	0.0442 (6)	
H1A	0.525 (2)	0.546 (4)	0.235 (3)	0.066*	
H1B	0.556 (3)	0.6725 (19)	0.209 (3)	0.066*	

### Atomic displacement parameters (Å^2^)

**Table:**

	*U*^11^	*U*^22^	*U*^33^	*U*^12^	*U*^13^	*U*^23^
Cl1	0.0513 (5)	0.0360 (4)	0.0371 (4)	−0.0030 (4)	0.0140 (4)	−0.0004 (3)
C1	0.0419 (18)	0.0288 (16)	0.0323 (16)	−0.0043 (14)	0.0180 (14)	−0.0020 (13)
C2	0.0445 (19)	0.0341 (18)	0.047 (2)	−0.0023 (15)	0.0157 (16)	−0.0088 (15)
C3	0.0415 (19)	0.0288 (16)	0.0351 (17)	−0.0036 (14)	0.0090 (15)	−0.0034 (13)
C4	0.0428 (18)	0.0371 (18)	0.0269 (16)	−0.0023 (14)	0.0043 (14)	−0.0020 (13)
C5	0.0401 (18)	0.0248 (15)	0.0344 (17)	−0.0064 (13)	0.0090 (14)	−0.0005 (12)
C6	0.0362 (17)	0.0407 (18)	0.0309 (17)	0.0022 (14)	0.0040 (14)	0.0005 (14)
C7	0.046 (2)	0.046 (2)	0.0277 (17)	−0.0003 (16)	0.0047 (15)	−0.0005 (14)
C8	0.0364 (18)	0.0312 (17)	0.0402 (18)	−0.0057 (14)	0.0059 (15)	−0.0048 (13)
C9	0.043 (2)	0.047 (2)	0.0370 (19)	0.0046 (16)	0.0062 (15)	0.0041 (15)
C10	0.048 (2)	0.055 (2)	0.0267 (17)	0.0061 (17)	0.0065 (15)	0.0049 (15)
C11	0.0412 (19)	0.046 (2)	0.042 (2)	0.0009 (16)	0.0138 (16)	−0.0027 (16)
C12	0.063 (4)	0.099 (6)	0.059 (4)	0.001 (4)	0.019 (3)	−0.028 (4)
C12'	0.127 (18)	0.048 (10)	0.19 (2)	−0.011 (11)	0.117 (18)	−0.025 (12)
C13	0.059 (4)	0.091 (5)	0.073 (5)	0.039 (4)	0.030 (3)	0.012 (4)
C13'	0.047 (10)	0.22 (3)	0.065 (12)	−0.012 (14)	0.020 (9)	−0.004 (16)
C14	0.059 (4)	0.063 (4)	0.105 (6)	−0.009 (3)	0.045 (4)	0.004 (4)
C14'	0.099 (14)	0.085 (13)	0.071 (11)	0.030 (11)	0.054 (10)	0.024 (9)
C15	0.042 (2)	0.0412 (19)	0.047 (2)	0.0041 (15)	0.0108 (17)	0.0045 (16)
C16	0.0409 (19)	0.0320 (17)	0.0409 (19)	0.0025 (14)	0.0090 (15)	−0.0021 (14)
C17	0.0345 (19)	0.084 (3)	0.040 (2)	0.0040 (19)	0.0053 (16)	−0.0035 (19)
C18	0.040 (2)	0.084 (3)	0.0345 (19)	0.005 (2)	0.0060 (16)	−0.0025 (19)
C19	0.0384 (19)	0.0323 (17)	0.0443 (19)	0.0036 (14)	0.0082 (15)	−0.0051 (14)
C20	0.038 (2)	0.076 (3)	0.048 (2)	0.004 (2)	0.0007 (17)	0.011 (2)
C21	0.050 (2)	0.074 (3)	0.040 (2)	0.004 (2)	0.0046 (18)	0.0118 (19)
C22	0.041 (2)	0.041 (2)	0.058 (2)	0.0041 (16)	0.0158 (18)	0.0008 (17)
C23	0.062 (3)	0.066 (3)	0.062 (3)	−0.009 (2)	0.023 (2)	0.007 (2)
C24	0.080 (3)	0.052 (3)	0.109 (4)	0.010 (2)	0.058 (3)	−0.001 (3)
C25	0.045 (2)	0.069 (3)	0.079 (3)	−0.010 (2)	0.007 (2)	0.012 (2)
N1	0.0390 (15)	0.0285 (13)	0.0340 (14)	−0.0035 (11)	0.0107 (12)	−0.0051 (11)
N2	0.0404 (16)	0.0305 (14)	0.0373 (15)	−0.0010 (12)	0.0130 (12)	−0.0010 (11)
O1	0.0508 (15)	0.0368 (13)	0.0481 (15)	−0.0018 (12)	0.0171 (12)	0.0024 (11)

### Geometric parameters (Å, °)

**Table:**

C1—N2	1.312 (4)	C13—H13C	0.9600
C1—N1	1.319 (4)	C13'—H13D	0.9600
C1—H1	0.9600	C13'—H13E	0.9600
C2—N2	1.475 (4)	C13'—H13F	0.9600
C2—C3	1.536 (5)	C14—H14A	0.9600
C2—H2A	0.9600	C14—H14B	0.9600
C2—H2B	0.9600	C14—H14C	0.9600
C3—N1	1.463 (4)	C14'—H14D	0.9600
C3—H3A	0.9600	C14'—H14E	0.9600
C3—H3B	0.9600	C14'—H14F	0.9600
C4—N1	1.458 (4)	C15—N2	1.449 (4)
C4—C5	1.511 (5)	C15—C16	1.506 (5)
C4—H4A	0.9600	C15—H15A	0.9600
C4—H4B	0.9600	C15—H15B	0.9600
C5—C10	1.383 (5)	C16—C21	1.365 (5)
C5—C6	1.398 (5)	C16—C17	1.381 (5)
C6—C7	1.380 (5)	C17—C18	1.386 (6)
C6—H6	0.9600	C17—H17	0.9600
C7—C8	1.392 (5)	C18—C19	1.383 (5)
C7—H7	0.9600	C18—H18	0.9600
C8—C9	1.392 (5)	C19—C20	1.372 (5)
C8—C11	1.539 (5)	C19—C22	1.538 (5)
C9—C10	1.387 (5)	C20—C21	1.393 (6)
C9—H9	0.9600	C20—H20	0.9600
C10—H10	0.9600	C21—H21	0.9600
C11—C12	1.497 (7)	C22—C23	1.525 (6)
C11—C12'	1.498 (16)	C22—C24	1.534 (6)
C11—C13'	1.517 (18)	C22—C25	1.535 (6)
C11—C14	1.532 (7)	C23—H23A	0.9599
C11—C13	1.544 (7)	C23—H23B	0.9599
C11—C14'	1.567 (15)	C23—H23C	0.9599
C12—H12A	0.9600	C24—H24A	0.9599
C12—H12B	0.9600	C24—H24B	0.9599
C12—H12C	0.9600	C24—H24C	0.9599
C12'—H12D	0.9600	C25—H25A	0.9599
C12'—H12E	0.9600	C25—H25B	0.9599
C12'—H12F	0.9600	C25—H25C	0.9599
C13—H13A	0.9600	O1—H1A	0.85 (3)
C13—H13B	0.9600	O1—H1B	0.839 (18)
			
N2—C1—N1	113.4 (3)	H13D—C13'—H13E	109.5
N2—C1—H1	123.3	C11—C13'—H13F	109.5
N1—C1—H1	123.3	H13D—C13'—H13F	109.5
N2—C2—C3	102.7 (3)	H13E—C13'—H13F	109.5
N2—C2—H2A	111.2	C11—C14—H14A	109.5
C3—C2—H2A	111.2	C11—C14—H14B	109.5
N2—C2—H2B	111.2	H14A—C14—H14B	109.5
C3—C2—H2B	111.2	C11—C14—H14C	109.5
H2A—C2—H2B	109.1	H14A—C14—H14C	109.5
N1—C3—C2	103.1 (3)	H14B—C14—H14C	109.5
N1—C3—H3A	111.1	C11—C14'—H14D	109.5
C2—C3—H3A	111.1	C11—C14'—H14E	109.5
N1—C3—H3B	111.1	H14D—C14'—H14E	109.5
C2—C3—H3B	111.1	C11—C14'—H14F	109.5
H3A—C3—H3B	109.1	H14D—C14'—H14F	109.5
N1—C4—C5	114.2 (3)	H14E—C14'—H14F	109.5
N1—C4—H4A	108.7	N2—C15—C16	111.9 (3)
C5—C4—H4A	108.7	N2—C15—H15A	109.2
N1—C4—H4B	108.7	C16—C15—H15A	109.2
C5—C4—H4B	108.7	N2—C15—H15B	109.2
H4A—C4—H4B	107.6	C16—C15—H15B	109.2
C10—C5—C6	117.9 (3)	H15A—C15—H15B	107.9
C10—C5—C4	119.2 (3)	C21—C16—C17	117.6 (4)
C6—C5—C4	122.8 (3)	C21—C16—C15	121.7 (3)
C7—C6—C5	120.4 (3)	C17—C16—C15	120.7 (3)
C7—C6—H6	119.8	C16—C17—C18	120.9 (4)
C5—C6—H6	119.8	C16—C17—H17	119.5
C6—C7—C8	122.5 (3)	C18—C17—H17	119.5
C6—C7—H7	118.8	C19—C18—C17	121.9 (4)
C8—C7—H7	118.8	C19—C18—H18	119.1
C9—C8—C7	116.2 (3)	C17—C18—H18	119.1
C9—C8—C11	122.7 (3)	C20—C19—C18	116.5 (4)
C7—C8—C11	121.1 (3)	C20—C19—C22	121.4 (3)
C10—C9—C8	122.0 (3)	C18—C19—C22	122.0 (3)
C10—C9—H9	119.0	C19—C20—C21	121.9 (4)
C8—C9—H9	119.0	C19—C20—H20	119.1
C5—C10—C9	121.0 (3)	C21—C20—H20	119.1
C5—C10—H10	119.5	C16—C21—C20	121.3 (4)
C9—C10—H10	119.5	C16—C21—H21	119.4
C12'—C11—C13'	116.1 (15)	C20—C21—H21	119.4
C12—C11—C14	109.9 (5)	C23—C22—C24	109.3 (4)
C12—C11—C8	111.1 (4)	C23—C22—C25	107.7 (3)
C12'—C11—C8	107.4 (8)	C24—C22—C25	109.3 (4)
C13'—C11—C8	109.9 (8)	C23—C22—C19	111.8 (3)
C14—C11—C8	108.1 (4)	C24—C22—C19	107.7 (3)
C12—C11—C13	108.1 (5)	C25—C22—C19	111.1 (3)
C14—C11—C13	107.1 (5)	C22—C23—H23A	109.5
C8—C11—C13	112.6 (4)	C22—C23—H23B	109.5
C12'—C11—C14'	107.6 (13)	H23A—C23—H23B	109.5
C13'—C11—C14'	104.3 (12)	C22—C23—H23C	109.5
C8—C11—C14'	111.6 (7)	H23A—C23—H23C	109.5
C11—C12—H12A	109.5	H23B—C23—H23C	109.5
C11—C12—H12B	109.5	C22—C24—H24A	109.5
H12A—C12—H12B	109.5	C22—C24—H24B	109.5
C11—C12—H12C	109.5	H24A—C24—H24B	109.5
H12A—C12—H12C	109.5	C22—C24—H24C	109.5
H12B—C12—H12C	109.5	H24A—C24—H24C	109.5
C11—C12'—H12D	109.5	H24B—C24—H24C	109.5
C11—C12'—H12E	109.5	C22—C25—H25A	109.5
H12D—C12'—H12E	109.5	C22—C25—H25B	109.5
C11—C12'—H12F	109.5	H25A—C25—H25B	109.5
H12D—C12'—H12F	109.5	C22—C25—H25C	109.5
H12E—C12'—H12F	109.5	H25A—C25—H25C	109.5
C11—C13—H13A	109.5	H25B—C25—H25C	109.5
C11—C13—H13B	109.5	C1—N1—C4	124.5 (3)
H13A—C13—H13B	109.5	C1—N1—C3	110.2 (3)
C11—C13—H13C	109.5	C4—N1—C3	124.1 (3)
H13A—C13—H13C	109.5	C1—N2—C15	126.6 (3)
H13B—C13—H13C	109.5	C1—N2—C2	110.1 (3)
C11—C13'—H13D	109.5	C15—N2—C2	122.9 (3)
C11—C13'—H13E	109.5	H1A—O1—H1B	120 (5)
			
N2—C2—C3—N1	−7.2 (3)	C15—C16—C17—C18	179.9 (4)
N1—C4—C5—C10	−155.8 (3)	C16—C17—C18—C19	0.7 (7)
N1—C4—C5—C6	28.6 (4)	C17—C18—C19—C20	−1.2 (6)
C10—C5—C6—C7	−0.8 (5)	C17—C18—C19—C22	−178.0 (4)
C4—C5—C6—C7	174.8 (3)	C18—C19—C20—C21	0.8 (6)
C5—C6—C7—C8	−0.8 (5)	C22—C19—C20—C21	177.6 (4)
C6—C7—C8—C9	1.1 (5)	C17—C16—C21—C20	−0.7 (6)
C6—C7—C8—C11	−178.1 (3)	C15—C16—C21—C20	179.7 (4)
C7—C8—C9—C10	0.2 (5)	C19—C20—C21—C16	0.2 (7)
C11—C8—C9—C10	179.3 (3)	C20—C19—C22—C23	156.2 (4)
C6—C5—C10—C9	2.1 (5)	C18—C19—C22—C23	−27.1 (5)
C4—C5—C10—C9	−173.7 (3)	C20—C19—C22—C24	−83.8 (5)
C8—C9—C10—C5	−1.8 (6)	C18—C19—C22—C24	92.9 (5)
C9—C8—C11—C12	134.6 (5)	C20—C19—C22—C25	35.9 (5)
C7—C8—C11—C12	−46.3 (6)	C18—C19—C22—C25	−147.4 (4)
C9—C8—C11—C12'	77.5 (13)	N2—C1—N1—C4	−170.4 (3)
C7—C8—C11—C12'	−103.5 (13)	N2—C1—N1—C3	−2.6 (4)
C9—C8—C11—C13'	−49.7 (13)	C5—C4—N1—C1	−120.4 (3)
C7—C8—C11—C13'	129.4 (13)	C5—C4—N1—C3	73.4 (4)
C9—C8—C11—C14	−104.8 (5)	C2—C3—N1—C1	6.3 (3)
C7—C8—C11—C14	74.3 (5)	C2—C3—N1—C4	174.2 (3)
C9—C8—C11—C13	13.3 (6)	N1—C1—N2—C15	−175.2 (3)
C7—C8—C11—C13	−167.7 (5)	N1—C1—N2—C2	−2.6 (4)
C9—C8—C11—C14'	−164.9 (9)	C16—C15—N2—C1	−121.4 (4)
C7—C8—C11—C14'	14.2 (10)	C16—C15—N2—C2	66.8 (4)
N2—C15—C16—C21	−130.9 (4)	C3—C2—N2—C1	6.2 (4)
N2—C15—C16—C17	49.5 (5)	C3—C2—N2—C15	179.2 (3)
C21—C16—C17—C18	0.3 (6)		

### Hydrogen-bond geometry (Å, °)

**Table:**

*D*—H···*A*	*D*—H	H···*A*	*D*···*A*	*D*—H···*A*
O1—H1A···Cl1^i^	0.86 (4)	2.36 (4)	3.206 (3)	174 (4)
C3—H3B···O1^ii^	0.96	2.53	3.304 (4)	138
C17—H17···O1^i^	0.96	2.47	3.423 (5)	175

Symmetry codes: (i) −*x*+1, −*y*+1, −*z*+1; (ii) −*x*+1, *y*−1/2, −*z*+1/2.
